# Dietary sanguinarine supplementation improves the growth performance and intestinal immunity of broilers

**DOI:** 10.1016/j.aninu.2024.05.009

**Published:** 2024-08-12

**Authors:** Yue Su, Guanyu Chang, Jingyu Liu, Peng Huang, Jianguo Zeng

**Affiliations:** a College of Veterinary Medicine, Shanxi Agricultural University, Taigu, Shanxi 030801, China; b College of Veterinary, Hunan Agricultural University, Changsha, Hunan 410128, China

**Keywords:** Sanguinarine, Intestinal immunity, Gut microbiota, Growth performance, Broiler

## Abstract

Dietary sanguinarine (SAN) can enhance the growth performance of poultry and livestock, but the regulatory mechanism of the SAN monomer on intestinal homeostasis and how it promotes growth performance has not yet been clarified. In this study, 200 chickens were divided into four groups and fed different doses of SAN (0, 0.225, 0.75, 2.25 mg/kg) for transcriptome and microbiota analysis. The data showed that different doses of SAN supplementation increased the feed conversion rate (FCR) of 22 to 42 d old and 1 to 42 d old broilers (*P* < 0.01), and 0.225 mg/kg SAN reduced the contents of alanine aminotransferase (ALT), aspartate aminotransferase (AST), creatinine (CREA) and blood urea nitrogen (BUN) in serum (*P* < 0.01). Dietary SAN increased the villus height and the villus height/crypt depth (V/C) ratio in the ileum (*P* < 0.01). The levels of tight junction proteins (zonula occludens-1, occludin and claudin*-*1) were up-regulated in the ileum and cecum (*P* < 0.01) and the levels of immunoglobulin (Ig) A, IgM, IgG, interleukin (IL)-4, IL-10 and interferon (IFN)-γ were up-regulated in the serum and ileum (*P* < 0.01). RNA-seq analysis revealed 385 differentially expressed genes (DEGs) (|log_2_ fold change| ≥ 1, FDR < 0.05) between the SAN group and CON group. Kyoto Encyclopedia of Genes and Genomes pathway analysis showed 15 pathways mostly associated with the immune system. Additionally, the reverse transcription-PCR results showed that the relative mRNA expression of β-defensin and mucin 2 were up-regulated (*P* < 0.01) and Toll-like receptor (*TLR2* and *TLR4*) mRNA expression were down-regulated by SAN (*P* < 0.01), which was consistent with the transcriptomic analysis. Western blot analysis also showed that SAN reduced the expression of inflammatory proteins such as TLR4, nuclear factor-kappa B and IL-1β in the ileum (*P* < 0.01). In addition, at the genus level, SAN significantly increased the relative abundance of bacteria (*Bacteroides*, *unclassified_f__Lachnospiraceae*, *Lactobacillus* and *Romboutsia*) involved in acetate and butyrate production in the cecum, which are associated with enhanced intestinal immune function and maintaining intestinal health. In conclusion, SAN ameliorates the growth performance of broilers, enhances intestinal immune function, regulates the structure of microbiota and maintains intestinal health.

## 1 Introduction

With the promotion of intensive farming techniques, various adverse factors have emerged in high-density animal farming systems, such as nutritional deficiencies, harsh environments and diseases, which cause high disease prevalence, high mortality for animals and significant economic losses for livestock farming (Jahanian and Khalifeh-Gholi, 2018; Sumanu et al., 2022; Zhou et al., 2023). To improve the productivity of livestock and poultry and prevent the occurrence of disease, feed additives such as antibiotic growth promoters (AGPs) have been extensively utilized (Lin et al., 2013). The improper utilization of AGPs not only aggravates the issue of antibiotic resistance, but also increases the accumulation of antibiotic residues in animal products. It's worth noting that antibiotic residues are subsequently excreted into the environment and pose a significant risk to public health (Chen et al., 2022; Suresh et al., 2018). Therefore, AGPs have been banned or have been subject to lower usage in many jurisdictions around the world. However, this ban could lead to a decline in the production efficiency and disease resistance of livestock and poultry (Mo et al., 2017). Consequently, the development of safe and effective substitutes for AGPs is of pressing importance to improve growth performance and disease resistance in these animals.

*Macleaya cordata* is a perennial herb indigenous to China, North America and Europe which alleviates inflammation, enhances growth and modifies gut microbiota (Huang et al., 2018; Khadem et al., 2014; Lin et al., 2018; Liu et al., 2013). In China, *M. cordata* extract (MCE) has been approved as a Chinese herbal medicine for animal feed, suitable for prolonged inclusion in animal diets (Li et al., 2018). A thorough understanding of the bioactive properties and regulatory mechanisms of MCE is paramount to unlocking its full therapeutic potential. This requires focused research on the active monomeric components of MCE, primarily the alkaloids of sanguinarine (SAN) and chelerythrine (Qing et al., 2021). Investigation of both the individual and combined effects of these alkaloids is essential to gain a nuanced understanding of how MCE exerts its biological activities. Intestinal inflammation can disrupt the gut barrier, leading to an imbalance in gut microbiota and a decline in growth performance (Satokari, 2015). It has been observed that the addition of 150 mg/kg *M. cordata* extract (Sangrovit) to the diet enhanced feed intake and body weight gain in broilers, while repairing damage in the duodenum, jejunum and ileum (Xue et al., 2017). Additionally, adding probiotics to feed has been shown to positively modulate gut microbiota structure and enhance broiler growth performance (Grozina et al., 2023). Previous research has found that dietary MCE can regulate the gut microbiota in broilers. It promotes the proliferation of Lactobacillus, restrains the colonization of *Escherichia coli*, activates the biosynthesis pathways of amino acids, vitamins and secondary bile acids, and decreases the accumulation of antibiotic resistant genes (Huang et al., 2018). Nevertheless, the mechanisms underlying SAN's influence on gut microbiota remain to be fully understood.

In this experiment, we supplied SAN at a dosage equivalent to that found in MCE (as determined from previous studies) in chicken feed to explore the effect of SAN on growth performance, immune function and intestinal microbiota. Our study elucidates the connection between gut immunity and microbiota via microbiome and transcriptome analysis and puts forward the potential regulatory mechanism of SAN on immune functionality in broiler chickens from the perspective of the modulation of gut microbiota composition and abundance.

## 2 Materials and methods

### 2.1 Animal ethics statement

The research adhered to animal experimentation ethics standards and received approval from the Research Ethics Committee of the College of Veterinary Medicine, Hunan Agricultural University (433320027).

### 2.2 Experimental material

The SAN (purity ≥ 98%) used in this experiment was supplied by Hunan Micolta Bioresource Co., Ltd., China, with batch number 140708.

### 2.3 Experimental design

A total of 200 broiler chickens with comparable body weights were chosen for this experiment, all of which were divided equally between males and females, and purchased from Hunan Shuncheng Industrial Co., Ltd., China. They were randomly divided into 4 groups, with 5 replicates and 10 chickens per replicate. The groups were as follows: CON group (basal diet), Low group (basal diet containing 0.225 mg/kg SAN), Middle group (basal diet containing 0.75 mg/kg SAN), and High group (basal diet containing 2.25 mg/kg SAN). The experimental period lasted for a duration of 42 d in its entirety.

The formulation of the basal diet used in this experiment followed the reference of China National Feeding Standard of Chicken (NY/T 33-2004), and the specific ingredients and nutritional levels can be found in Table 1. To determine the composition of the diet, the contents of crude protein and crude lipid in feed samples were measured by using Kjeldahl nitrogen determination and Soxhlet extraction according to China National Standards (GB/T 6432-2018 and GB/T 6433-2006), respectively. The values of metabolizable energy (ME) was calculated by referring to ME of each feed material provided in the China Feed Database (2020).

**Table 1:** Ingredients and nutrient compositions of the experimental control diet (%, as-fed basis).

Item	Content
Ingredients	
Corn	61.00
Soybean meal	30.00
Wheat	
Soybean oil	5.20
Stone powder	1.10
Methionine	0.13
L-Lysine	0.30
CaHPO_4_	1.67
Premix^1^	0.60
Total	100.00
Nutrient compositions^2^	
Metabolizable energy, MJ/kg	11.86
Crude protein	18.79
Crude lipid	19.84

1 Provided the following per each kilogram of full-price diet: 25% copper sulfate pentahydrate, 70 mg; 30% ferrous sulfate water, 150 mg; 35% zinc sulfate monohydrate, 300 mg; 31% manganese sulfate monohydrate, 400 mg; 1% selenium, 50 mg; 1% iodine, 150 mg; multidimensional, 300 mg; choline, 500 mg; antioxidant, 100 mg; zeolite powder, 400 mg; fine bran, 380 mg; phytase, 200 mg; salt, 3000 mg.

2 Metabolizable energy was calculated value and the others were measured values.

### 2.4 Growth performance

Upon concluding each respective experimental phase (21 and 42 d), fasting was performed for 12 h (with free access to water). Individual weights of each group were measured, and feed intake of each group was documented at various stages throughout the experiment. Based on these data, average daily feed intake (ADFI), average daily gain (ADG) and feed conversion rate (FCR) were calculated for each group.

### 2.5 Sample collection

At the conclusion of the experiment, 5 chickens with similar average body weight from each group were chosen at random. After fasting for 12 h, blood samples were obtained from the jugular vein, centrifuged at 3000 × *g* for 15 min at 4 °C. The resulting serum was collected and stored at −20 °C until subsequent analysis of experimental indices. Ileal tissues were collected and preserved using 4% paraformaldehyde. The contents of the cecum were collected in 2-mL Eppendorf tubes, snap frozen in liquid nitrogen, and subsequently transferred to −80 °C for storage until further analysis of the cecal microbiota using 16S rRNA gene amplicon sequencing. The remaining tissues of the ileum and cecum were washed, and frozen in liquid nitrogen, then transferred to −80 °C for storage.

### 2.6 Measurement of intestinal morphology

The fixed ileal tissue underwent dehydration and paraffin embedding. The sections were stained with hematoxylin and eosin (H&E) and evaluated under a light microscope. The height of the ileal villi and corresponding crypt depths were measured using CaseViewer software. The ratio of villus height to crypt depth was calculated. Each sample was sliced in duplicate, and five fields per slice were randomly selected for statistical analysis.

### 2.7 Serum biochemical parameters

The experiment utilized the Mindray BS-200 fully automated biochemistry analyzer and matching reagent kits manufactured by KHB Bio-Tech Co., Ltd. to measure serum alanine aminotransferase (ALT), aspartate aminotransferase (AST), alkaline phosphatase (ALP), total protein (TP), albumin (ALB), glucose (GLU), creatinine (CREA), blood urea nitrogen (BUN), uric acid (UA), creatine kinase (CK), and lactate dehydrogenase (LDH).

### 2.8 Cytokines and immunoglobulins

Enzyme-linked immunosorbent assay (ELISA) kits were utilized to measure the serum and ileal concentrations of interleukin-4 (IL-4), interleukin-10 (IL-10), interferon-gamma (IFN-γ), immunoglobulin A (IgA), immunoglobulin M (IgM), and immunoglobulin G (IgG).

### 2.9 Transcriptome sequencing analysis

TRIzol reagent was used to extract total RNA from ileal samples of the CON and Low groups (*n =* 4), which was then assessed for concentration and purity using the Nanodrop2000. The RNA quality number (RQN) was calculated using the Agilent 5300 Bioanalyzer. For library construction, the minimum requirement for total RNA was greater or equal to 1 μg, with a concentration greater or equal to 35 ng/μL, an optical density (OD)_260/280_ ratio greater or equal to 1.8, and an OD_260/230_ ratio greater or equal to 1.0. Subsequently, the Illumina NovaSeq 6000 platform was used to sequence short sequence fragments. Differentially expressed genes (DEGs) were identified as those with *P* ≤ 0.05 and log_2_ (fold change) ≥ 1. Functional enrichment analysis was conducted using gene ontology (GO) and Kyoto Encyclopedia of Genes and Genomes (KEGG) to determine enriched DEGs.

### 2.10 Reverse transcription-PCR (RT-PCR)

TRIzol reagent was used to extract total RNA from ileal and cecal tissue samples, and the concentration of RNA was measured using a microplate spectrophotometer. Reverse transcription was performed to synthesize cDNA, following the instructions of Transcriptor cDNA Synth (Accurate Biotechnology (Hunan) Co., Ltd., China). Following this, 20 ng of cDNA was used in each PCR reaction with a reaction volume of 10 μL. The qTOWER3 G instrument was used to perform real-time fluorescence quantitative PCR analysis with SYBR Green I Master Mix. The relative expression levels of targeted genes were determined utilizing the 2^−ΔΔC^^t^ method, with the reference gene being β-actin. The primers used for amplification were synthesized by Sangon Biotech Co., Ltd (Shanghai, China) and listed in Table S1.

### 2.11 Western blot analysis

Once the total protein was extracted from the chicken ileum, protein samples were prepared based on their concentration determined by the bicinchoninic acid (BCA) method. Sodium dodecyl sulfate-polyacrylamide gel electrophoresis (SDS-PAGE) was used to separate the target proteins, which were then transferred onto a polyvinylidene fluoride (PVDF) membrane. Following this, the membrane was blocked with 5% milk for 90 min at room temperature. The primary antibodies against zonula occludens-1 (ZO-1), nuclear factor-kappa B (NF-κB), Toll-like receptor 4 (TLR4), and interleukin-1β (IL-1β) (Proteintech Group, Inc.) were added and incubated overnight at 4 °C. After washing with Tris-buffered saline with Tween 20 (TBST), the PVDF membranes were incubated with appropriate secondary antibodies for 50 min. Finally, enhanced chemiluminescent reagents were used for visualization. The grayscale values of the target protein bands were determined using Image J software. Grayscale values of bands were normalized against their respective controls. Three independent experiments were performed as biological replicates.

### 2.12 16S rRNA gene amplicon sequencing

Microbial DNA was extracted from the cecal content samples (*n =* 5) using the OMG-Soil DNA Kit (Omega Bio-Tek, Georgi, USA). The purified and quantified DNA was determined by the QuantiFluor™ ST blue fluorescence quantitation system (Promega) and 1% agarose gel electrophoresis, respectively. The hypervariable region V3–V4 of the bacterial 16S rRNA gene were amplified with primer pairs 338F (5′- ACTCCTACGGGAGGCAGCAG-3′) and 806R (5′-GGACTACHVGGGTWTCTAAT-3′) by using the thermocycler PCR system (ABI GeneAmp&reg 9700). Then, the Illumina MiSeq platform was performed to generate paired-end sequencing libraries (2 × 300 bp) of purified amplicons (Majorbio Bio-Pharm Technology Co. Ltd., Shanghai, China). After the raw FASTQ files were de-multiplexed using an in-house Perl script, and quality-filtered by fastp version 0.19.6 and merged by FLASH version 1.2.7, optimized sequences were obtained. Using the optimized sequences, operational taxonomic units (OTUs) were clustered at a similarity level of 97% using the Uparse method, and the representative OTU sequences were used to obtain taxonomic information for bioinformatics analysis. Mothur software was utilized for α diversity analysis. Qiime software was employed to compute the β diversity distance matrix, and significant differences in microbes in different groups of chicken cecal microbiota were determined using the Kruskal_Wallis H test. R language tools were used for data visualization. To identify species exhibiting notable differences in abundance between groups, the linear discriminant analysis (LDA) effect size (LEfSe) approach was implemented. Correlations between gut microbiota and other parameters were assessed through Spearman's correlation analysis. All data analyses were executed on the Majorbio cloud platform (www.majorbio.com).

### 2.13 Short chain fatty acid (SCFA) content determination

Twenty cecal content samples, identical to those used for 16S rRNA sequencing, were selected for the determination of five SCFA concentrations, namely acetic acid, propionic acid, butyrate, isobutyric acid and valeric acid. Briefly, approximately 0.5 g of cecal contents were taken and added into 1-mL phosphate-buffered saline (PBS), vortexed for 30 min and left overnight. After centrifugation at 10,000 × *g* for 15 min at 4 °C, the supernatant was collected. Subsequently, a centrifuge tube was employed to combine 900 μL of the supernatant with 100 μL of 25% metaphosphoric acid. This mixture was centrifuged once more under identical conditions, and the resulting supernatant was then filtered using a 0.45- μm filter membrane. The SCFA content was calculated by gas chromatography (Shimadzu 2010 plus type gas chromatogram) using a DB-fused silica free fatty acid phase chromatographic (DB-FFAP) column of size 30 m × 0.25 mm × 0.25 μm. The carrier gas was high purity nitrogen (99.999%), the flow rate was 0.8 mL/min. The auxiliary gas was high purity hydrogen (99.999%), the flame ionization detector (FID) temperature of the detector was 280 °C, the temperature of the injection port was 250 °C, the split ratio was 50:1, and the injection volume was 1 μL. The temperature increase was programmed to commence at 60 °C, and rise at a rate of 20 °C/min until eventually reaching 220 °C. Subsequently, the temperature was sustained for 1 min.

### 2.14 Calculations and statistical analysis

The ME for this experiment was calculated using the following equation:

ME (MJ/kg) = corn × ME1 + wheat × ME2 + soybean meal × ME3.

The data provided by the China Feed Database was used to determine the ME1, ME2, and ME3 values for corn, wheat, and soybean meal, respectively.

The following index method equation was used to calculate crude protein:

$$\text{Crude}\;\text{protein}\;\left(\%\right)=\frac{\left(V-V_{0}\right)\times C\times 0.014\times 6.25}{M}\times 100$$

where *V* is the volume of hydrochloric acid in experimental group, *V*_0_ is the volume of hydrochloric acid in control group, *C* is the concentration of hydrochloric acid, and *M* is the quality of sample.

Crude lipid was calculated by the following equation:

$$\text{Crude}\;\text{lipid}\;\left(\%\right)=\frac{m_{1}-m_{2}}{m}\times 100$$

where m is the sample weight, *m*_1_ is the lipid weight after drying before serving, *m*_2_ is the lipid weight after extraction and drying.

Data analysis for each group was conducted using SPSS 27.0 software. One-way ANOVA was used to assess variations among groups, and multiple comparison tests were performed by the application of the Tukey–Kramer test. Linear and quadratic relationships were established. GraphPad Prism 8.0 was utilized to generate the corresponding plots. The correlations among SCFAs, immune-related factors and microbial structure were analyzed using Spearman's rank correlation analysis. The obtained outcomes are expressed as mean ± standard error of the mean (SEM). A value of *P* < 0.05 suggests statistical significance, while *P* < 0.01 indicates a highly significant difference.

## 3 Results

### 3.1 Effects of SAN on growth performance of broilers

As displayed in Table 2, during the experiment from d 1 to 21, compared with the CON group, the inclusion of different doses of SAN in the diet did not elicit any significant impact on the ADFI, ADG and FCR of broilers (*P* = 0.082, *P* = 0.286 and *P* = 0.741). However, in the subsequent study period between d 22 and 42, there was an increase in the ADG of broilers observed in SAN groups (*P* = 0.052), and the FCR values of all the SAN-treated groups were found to be lower than those of the CON group (*P* = 0.005). Throughout the entire feeding period, the inclusion of varying doses of SAN into the broiler diet resulted in an increase in ADG (*P* = 0.065), and the FCR in all SAN-supplemented groups showed a significant decrease compared to the CON group (*P* = 0.006).

**Table 2:** Effects of dietary sanguinarine (SAN) supplementation on growth performance of broilers.

Item	Group^1^				*P*-value		
	CON	Low	Middle	High	ANOVA	Linear	Quadratic
1–21 d							
ADFI, g	32.1 ± 1.14	31.3 ± 0.65	31.7 ± 0.42	34.3 ± 0.85	0.082	0.09	0.03
ADG, g	25.3 ± 0.93	24.8 ± 0.58	24.5 ± 0.47	26.3 ± 0.61	0.286	0.4	0.17
FCR, %	1.3 ± 0.05	1.3 ± 0.01	1.3 ± 0.01	1.3 ± 0.02	0.741	0.29	0.56
22–42 d							
ADFI, g	106.2 ± 5.26	108.5 ± 1.52	105.5 ± 1.14	111.9 ± 1.32	0.409	0.28	0.45
ADG, g	66.9 ± 4.06	77.8 ± 3.38	72.3 ± 1.26	76.9 ± 1.57	0.052	0.09	0.15
FCR, %	1.6 ± 0.03	1.4 ± 0.04∗∗	1.5 ± 0.03∗∗	1.5 ± 0.03∗∗	0.005	0.07	0.01
1–42 d							
ADFI, g	69.1 ± 2.80	69.9 ± 0.79	68.6 ± 0.67	73.1 ± 1.03	0.221	0.16	0.2
ADG, g	46.1 ± 2.27	51.3 ± 1.79	48.4 ± 0.57	51.6 ± 0.78	0.065	0.08	0.19
FCR, %	1.5 ± 0.01	1.4 ± 0.03∗∗	1.4 ± 0.02∗∗	1.4 ± 0.02∗∗	0.006	0.13	0.02

ADFI = average daily feed intake; ADG = average daily gain; FCR = feed conversion rate.

Values are expressed as mean ± SEM (*n =* 5). Compared with the CON group, ∗*P* < 0.05; ∗∗*P* < 0.01.

1 CON, control group (basal diet); Low, low-dose of sanguinarine group (basal diet containing 0.225 mg/kg sanguinarine); Middle, medium-dose of sanguinarine group (basal diet containing 0.75 mg/kg sanguinarine); High, high-dose of sanguinarine group (basal diet containing 2.25 mg/kg sanguinarine).

### 3.2 Effects of SAN on serum biochemical indices of broilers

As shown in Table 3, compared with the CON group, the Low group exhibited an extremely significant decrease in serum levels of ALT, CREA, BUN, CK, LDH (*P* < 0.001) and AST (*P* = 0.003). Additionally, the level of ALP was found to be down-regulated (*P* = 0.018), whereas the levels of TP and ALB were extremely significantly increased (*P* < 0.001). The Middle group had a highly significant decrease in the levels of ALT, ALP, UA, CREA, BUN, CK, and LDH (*P* < 0.001), while the contents of TP (*P* < 0.001) and ALB (*P* = 0.005) experienced a significant increase. Similarly, the High group demonstrated an extremely significant decrease in serum contents of ALT, ALP, UA, BUN and CK (*P* < 0.001), and an extremely significant increase in the levels of TP, ALB (*P* < 0.001) and CREA (*P* = 0.008).

**Table 3:** Effects of dietary sanguinarine (SAN) supplementation on serum biochemistry parameters of broilers.

Item	Group^1^				*P*-value		
	CON	Low	Middle	High	ANOVA	Linear	Quadratic
ALT, U/L	6.4 ± 0.22	4.7 ± 0.18∗∗	4.1 ± 0.22∗∗	4.4 ± 0.14∗∗	<0.001	<0.001	<0.001
AST, U/L	506.1 ± 25.27	418.1 ± 13.11∗∗	463.4 ± 6.68	497.1 ± 22.44	0.012	0.861	0.017
ALP, U/L	1461.1 ± 25.58	1370.9 ± 18.31∗	842.8 ± 33.23∗∗	1005.0 ± 18.46∗∗	<0.001	<0.001	<0.001
TP, g/L	31.7 ± 0.37	35.5 ± 0.50∗∗	35.7 ± 0.33∗∗	34.8 ± 0.60∗∗	<0.001	0.004	<0.001
ALB, g/L	11.2 ± 0.22	12.1 ± 0.12∗∗	11.9 ± 0.12∗∗	12.5 ± 0.20∗∗	<0.001	<0.001	<0.001
UA, μmol/L	214.7 ± 2.99	210.7 ± 4.23	153.8 ± 5.50∗∗	165.0 ± 3.45∗∗	<0.001	<0.001	<0.001
CREA, mol/L	4.0 ± 0.10	1.5 ± 0.01∗∗	1.9 ± 0.07∗∗	5.1 ± 0.49∗∗	<0.001	0.228	<0.001
BUN, mmol/L	0.4 ± 0.01	0.3 ± 0.01∗∗	0.3 ± 0.02∗∗	0.3 ± 0.01∗∗	<0.001	0.048	<0.001
GLU, mmol/L	11.4 ± 0.17	11.9 ± 0.26	11.2 ± 0.24	11.9 ± 0.13	0.091	0.511	0.723
CK, mol/L	15.6 ± 0.27	12.5 ± 0.34∗∗	13.5 ± 0.36∗∗	12.6 ± 0.23∗∗	<0.001	0.001	<0.001
LDH, mol/L	3.0 ± 0.04	2.6 ± 0.04∗∗	2.8 ± 0.02∗∗	2.9 ± 0.05	<0.001	0.656	<0.001

ALT = alanine aminotransferase; AST = aspartate aminotransferase; ALP = alkaline phosphatase; TP = total protein; ALB = albumin; GLU = glucose; CREA = creatinine; BU*N =* blood urea nitrogen; UA = uric acid; CK = creatine kinase; LDH = lactate dehydrogenase.

Values are expressed as mean ± SEM (*n =* 5). Compared with the CON group, ∗*P* < 0.05; ∗∗*P* < 0.01.

1 CON, control group (basal diet); Low, low-dose of sanguinarine group (basal diet containing 0.225 mg/kg sanguinarine); Middle, medium-dose of sanguinarine group (basal diet containing 0.75 mg/kg sanguinarine); High, high-dose of sanguinarine group (basal diet containing 2.25 mg/kg sanguinarine).

### 3.3 Effects of dietary SAN supplementation on intestinal barrier of broilers

**Fig. 1:**
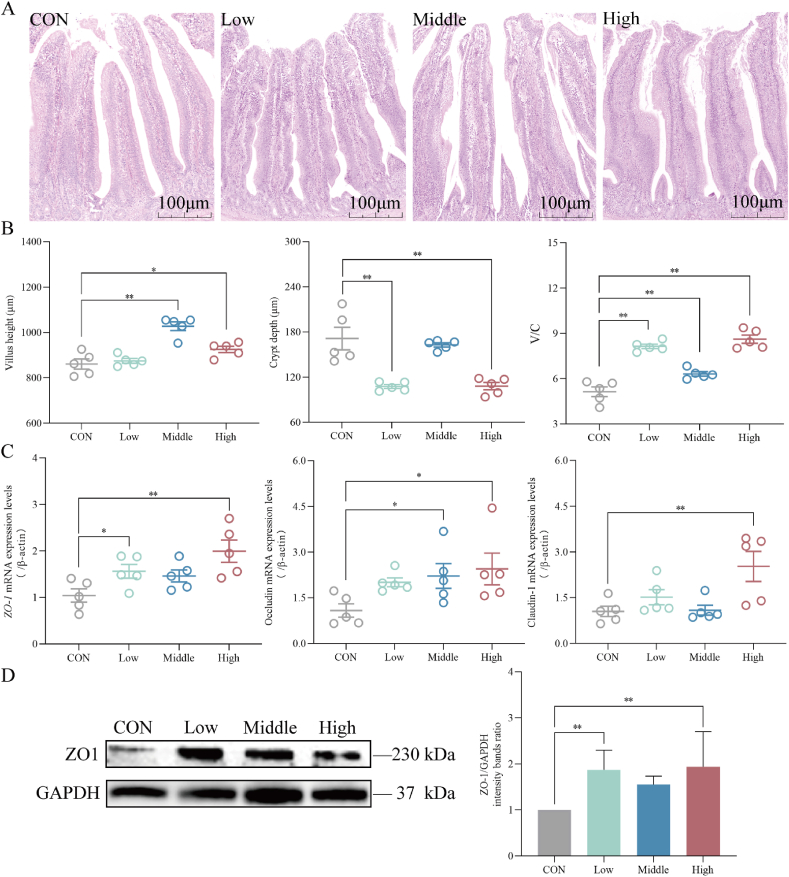
Effects of dietary sanguinarine (SAN) supplementation on the ileal barrier of broilers. (A) Optical microscopy was used to monitor hematoxylin and eosin (H&E) stained sections. (B) The villus height, crypt depth and villus height/crypt depth (V/C) of the ileum were analyzed using HE staining. (C) The mRNA expression levels of *ZO-1*, occludin and claudin-1 were examined using RT-PCR. (D) The relative expression of ZO-1 protein in the ileum was measured through Western blot analysis. ZO-1 = zonula occludens-1. GAPDH = glyceraldehyde-3-phosphate dehydrogenase. CON, control group (basal diet); Low, low-dose of SAN group (basal diet containing 0.225 mg/kg SAN); Middle, medium-dose of SAN group (basal diet containing 0.75 mg/kg SAN); High, high-dose of SAN group (basal diet containing 2.25 mg/kg SAN). Values are expressed as mean ± SEM (*n =* 5). ∗*P* < 0.05 and ∗∗*P* < 0.01.

As demonstrated in Fig. 1A and B, compared with the CON group, dietary different doses of SAN supplementation increased villus height in the ileum, particularly in the Middle group which showed an extremely significant difference (*P* < 0.01), while the High group displayed a significant difference relative to the CON group (*P* < 0.05). The Low and High groups exhibited a significant decrease in the crypt depth of the ileum (*P* < 0.01), resulting in an increase in the V/C ratio across all SAN treatment groups. As shown in Fig. 1C, the Low group demonstrated a significant increase in the mRNA level of *ZO-1* compared to the CON group (*P* < 0.05), and the mRNA level of occludin was increased in the Middle group (*P* < 0.05), while the mRNA levels of *ZO-1* and claudin-1 were up-regulated in the High group (*P* < 0.01), and the relative expression of occludin mRNA was also found to be increased (*P* < 0.05). As shown in Fig. 1D, different doses of dietary SAN supplementation could up-regulate the protein expression level of ZO-1 in ileal tissue, and the differences were extremely significant in the Low and High groups (*P* < 0.01).

### 3.4 Effects of SAN on serum cytokine and immunoglobulin levels in broilers

**Fig. 2:**
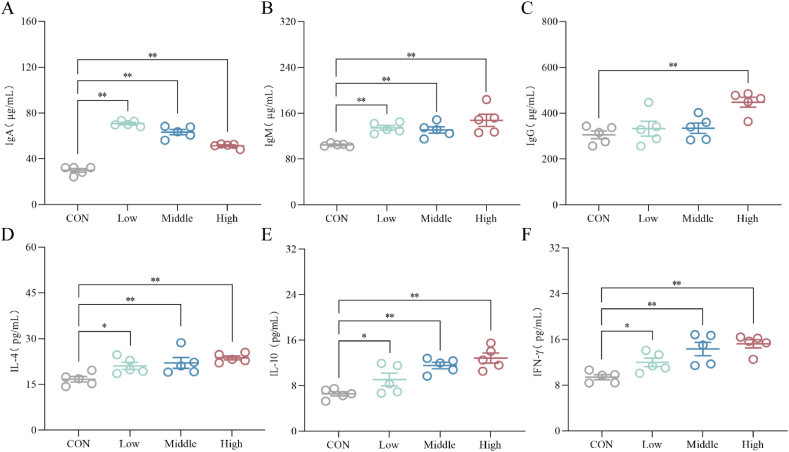
Effects of dietary sanguinarine (SAN) supplementation on the levels of immunoglobulins and cytokines in the serum of broilers. The levels of (A) IgA, (B) IgM, (C) IgG, (D) IL-4, (E) IL-10 and (F) IFN-γ in the serum were analyzed by ELISA kits. IL-4 = interleukin-4; IL-10 = Interleukin-10; IFN-γ = interferon-gamma; IgA = immunoglobulin A; IgM = immunoglobulin M; IgG = immunoglobulin G. CON, control group (basal diet); Low, low-dose of SAN group (basal diet containing 0.225 mg/kg SAN); Middle, medium-dose of SAN group (basal diet containing 0.75 mg/kg SAN); High, high-dose of SAN group (basal diet containing 2.25 mg/kg SAN). Values are expressed as mean ± SEM (*n =* 5). ∗*P* < 0.05 and ∗∗*P* < 0.01.

Fig. 2A–F demonstrated that the Low group of chickens exhibited a significant increase in IgA and IgM levels in serum (*P* < 0.01), as well as a significant increase in IL-4 and IFN-γ levels compared to the CON group (*P* < 0.05). The Middle group exhibited a significant increase in serum contents of IgA, IL-4, IL-10 and IFN-γ (*P* < 0.01), as well as a significant increase in IgM levels (*P* < 0.05). Additionally, the High group showed a significant increase in the concentration of IgG, IgM, IgA, IL-4, IL-10 and IFN-γ (*P* < 0.01).

### 3.5 Effects of SAN on ileal cytokine and immunoglobulin levels in broilers

**Fig. 3:**
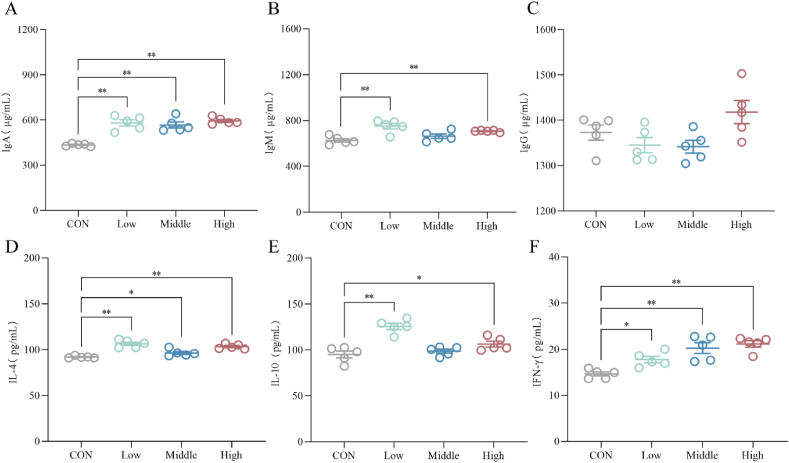
Effects of dietary sanguinarine (SAN) supplementation on the levels of immunoglobulins and cytokines in the ileum of broilers. The levels of (A) IgA, (B) IgM, (C) IgG, (D) IL-4, (E) IL-10 and (F) IFN-γ in the ileum were analyzed by ELISA kits. IL-4 = interleukin-4; IL-10 = interleukin-10; IFN-γ = interferon-gamma; IgA = immunoglobulin A; IgM = immunoglobulin M; IgG = immunoglobulin G. CON, control group (basal diet); Low, low-dose of SAN group (basal diet containing 0.225 mg/kg SAN); Middle, medium-dose of SAN group (basal diet containing 0.75 mg/kg SAN); High, high-dose of SAN group (basal diet containing 2.25 mg/kg SAN). Values are expressed as mean ± SEM (*n =* 5). ∗*P* < 0.05 and ∗∗*P* < 0.01.

As shown in Fig. 3A–F, the SAN-treated groups of broilers exhibited a significant increase in the content of IgA in ileal tissue compared to the CON group (*P* < 0.01). The Low group demonstrated a significant elevation in the levels of IgM, IL-4, and IL-10 (*P* < 0.01), while the IFN-γ level showed a significant increase (*P* < 0.05). The level of IFN-γ showed an extremely significant increase in the Middle group (*P* < 0.01), while the IL-4 level exhibited an increase (*P* < 0.05). In the High group, the IgM, IL-4 and IFN-γ levels were elevated (*P* < 0.01), while the IL-10 level showed a significant increase (*P* < 0.05).

### 3.6 Ileal transcriptomic analysis

**Fig. 4:**
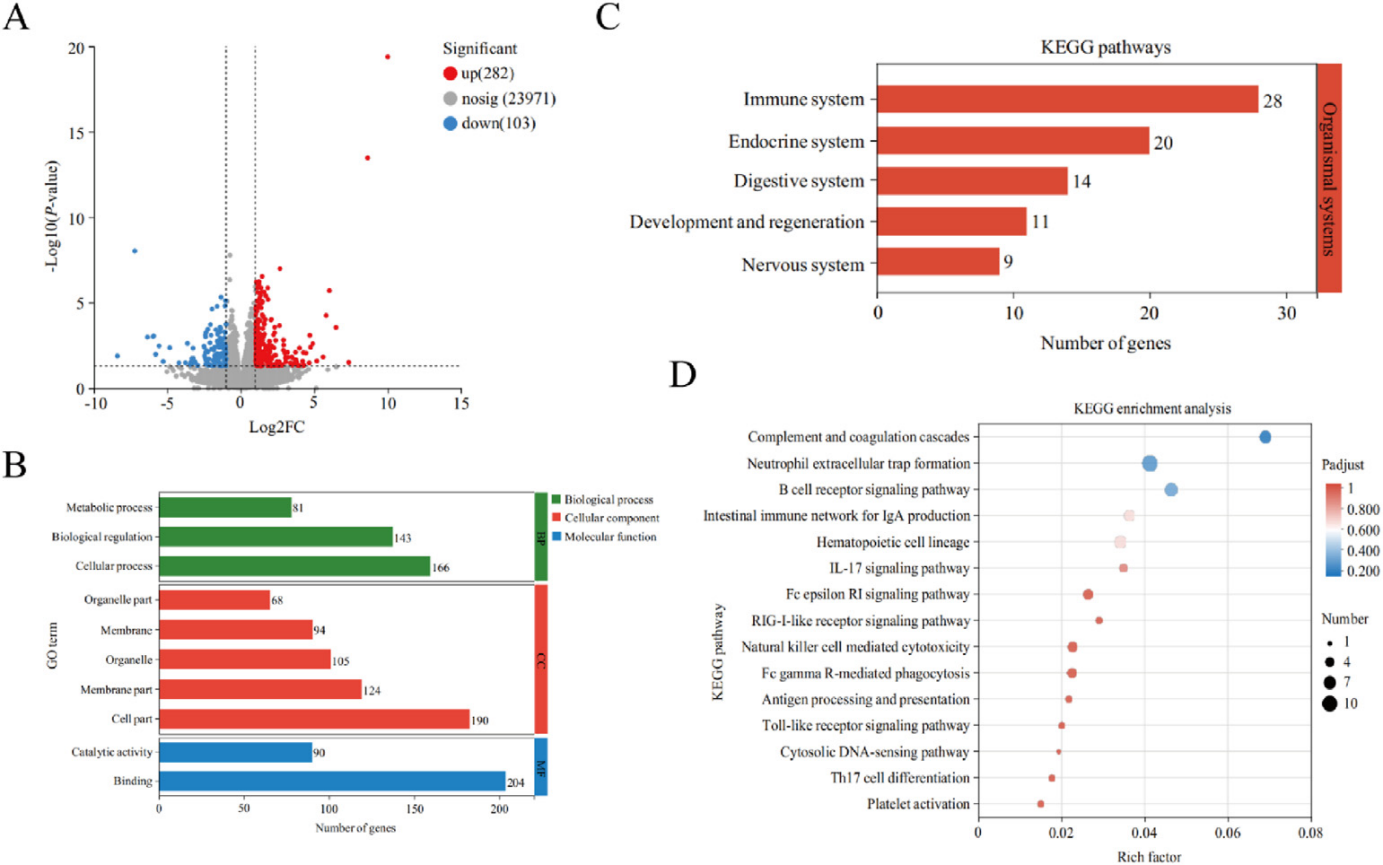
The modulatory effects of sanguinarine (SAN) on ileum transcriptomics (*n =* 4). (A) The volcano plot for the genes between CON and Low groups. (B) Gene ontology (GO) annotation analysis of differentially expressed genes (DEGs). (C) Kyoto Encyclopedia of Genes and Genomes (KEGG) annotation analysis of DEGs. (D) The KEGG pathway enrichment analysis of the differentially expressed genes.

Through transcriptome sequencing, we conducted additional analysis to investigate the impact of SAN on gene expression in the ileal tissue of broilers. As depicted in Fig. 4A, SAN regulated the expression of 385 genes in the ileum, with 282 genes up-regulated and 103 genes down-regulated. GO annotation was performed for all genes and differentially expressed genes as presented in Fig. 4B, the majority of genes were classified under the biological process category, particularly in cell processes and bioregulation. The cellular component category had the majority of genes annotated under the cell part subcategory. The binding term had the most genes annotated in the molecular function category. To classify the genes in the gene set based on their participating pathways or functions, we utilized the KEGG database. Among them, KEGG functional annotations related to organismal system are presented in Fig. 4C, and the majority of genes were annotated to immune system. As demonstrated in Fig. 4D, KEGG enrichment analysis of DEGs related to immune system was performed, and the top of 15 pathways included complement and coagulation cascades, neutrophil extracellular trap formation, B cell receptor signaling pathway, hematopoietic cell lineage, intestinal immune network for IgA production, IL-17 signaling pathway, Fc epsilon RI signaling pathway, retinoic acid-inducible gene I (RIG-I) -like receptor signaling pathway, Natural killer cell mediated cytotoxicity, Fc gamma R-mediated phagocytosis, antigen processing and presentation, Toll-like receptor signaling pathway, cytosolic DNA-sensing pathway, Th17 cell differentiation and platelet activation.

### 3.7 To verify the expression of immune-related genes in the ileum

**Fig. 5:**
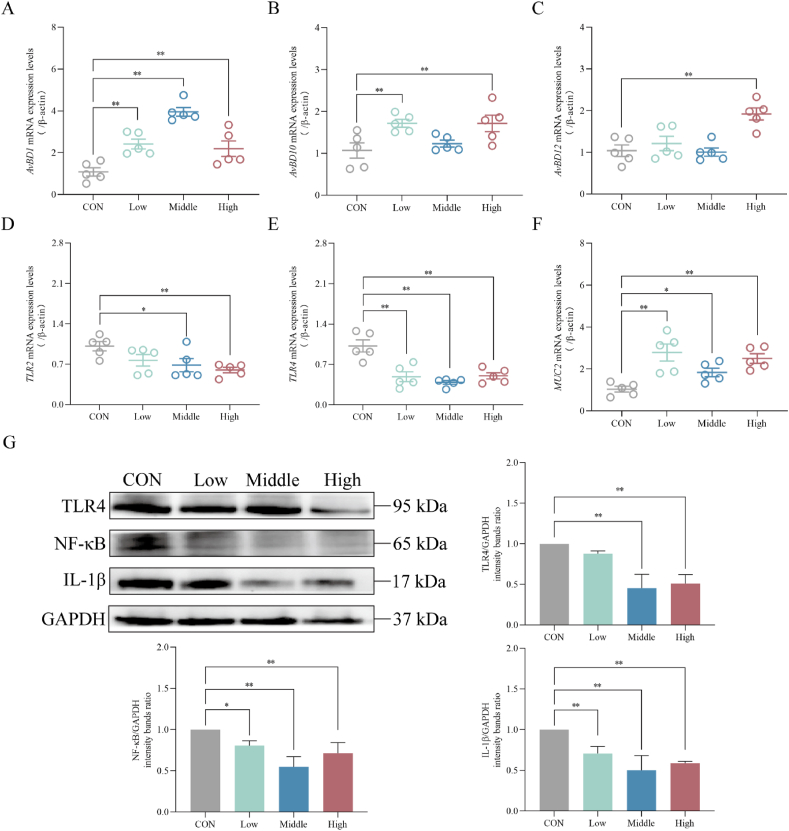
Effects of dietary sanguinarine (SAN) supplementation on the expression of ileal immune factors of broilers. The mRNA expression levels of (A) *AvBD1*, (B) *AvBD10*, (C) *AvBD12*, (D) *TLR2*, (E) *TLR4* and (F) *MUC2* in the ileum were analyzed by RT‒PCR. (G) The relative expression of TLR4, NF-κB and IL-1β protein in the ileum was measured by Western blot. *AvBD1* = avian beta-defensin 1; *AvBD10* = avian beta-defensin 10; *AvBD12* = avian beta-defensin 12; *MUC2* = mucin 2; *TLR2* = Toll-like receptor 2; *TLR4* = Toll-like receptor 4; *NF-κB* = nuclear factor-kappa B; GAPDH = glyceraldehyde-3-phosphate dehydrogenase. CON, control group (basal diet); Low, low-dose of SAN group (basal diet containing 0.225 mg/kg SAN); Middle, medium-dose of SAN group (basal diet containing 0.75 mg/kg SAN); High, high-dose of SAN group (basal diet containing 2.25 mg/kg SAN). Values are expressed as mean ± SEM (*n =* 5). ∗*P* < 0.05 and ∗∗*P* < 0.01.

We selected several up-regulated genes (avian beta-defensin [*AvBD1*, *AvBD10*, *AvBD12*] and mucin 2 [*MUC2*]) and down-regulated genes (*TLR2* and *TLR4*) for validation by RT-PCR. As shown in Fig. 5A–F, in comparison to the CON group, the expression levels of *AvBD1* were increased (*P* < 0.01), while *TLR4* was decreased (*P* < 0.01) following dietary supplementation with different doses of SAN. The Low group exhibited a significant increase in mRNA levels of *AvBD10* and *MUC2* (*P* < 0.01). In contrast, the Middle group displayed a significant increase in the content of MUC2 (*P* < 0.05), but *TLR2* expression showed a significant decrease (*P* < 0.05) in the ileal tissue of broilers. Within the High group, the mRNA expression levels of *AvBD10*, *AvBD12* and *MUC2* were increased (*P* < 0.01), while *TLR2* mRNA levels were decreased in the ileal tissue (*P* < 0.01). Western blot was conducted to assess the protein expression of *TLR4*, *NF-κB* and *IL-1β* in ileal tissue to gauge the impact of SAN on intestinal immunity. The results illustrated in Fig. 5G established that compared to the CON group, the Low group exhibited down-regulated inflammatory protein expression of IL-1β (*P* < 0.01) and NF-κB (*P* < 0.05) within the ileum. Similarly, the ileal tissue of both the Middle and High groups exhibited down-regulated expression levels of TLR4, NF-κB and IL-1β (*P* < 0.01).

### 3.8 Effects of SAN on expression of immune-related genes in the cecum of broilers

**Fig. 6:**
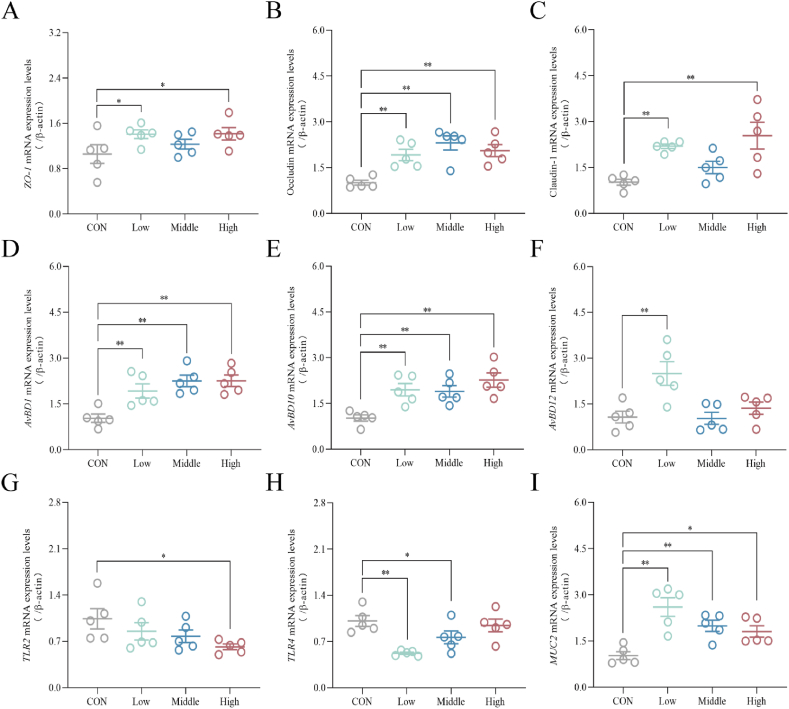
Effects of dietary sanguinarine (SAN) supplementation on the expression of cecal immune factors in broilers. The mRNA expression levels of (A) *ZO-1*, (B) occludin, (C) claudin-1, (D) *AvBD1*, (E) *AvBD10*, (F) *AvBD12*, (G) *TLR2*, (H) *TLR4* and (I) *MUC2* in the cecum were analyzed by RT‒PCR. *ZO-1* = zonula occludens-1; *AvBD1* = avian beta-defensin 1; *AvBD10* = avian beta-defensin 10; *AvBD12* = avian beta-defensin 12; *MUC2* = mucin 2; *TLR2* = Toll-like receptor 2; *TLR4* = Toll-like receptor 4. CON, control group (basal diet); Low, low-dose of SAN group (basal diet containing 0.225 mg/kg SAN); Middle, medium-dose of SAN group (basal diet containing 0.75 mg/kg SAN); High, high-dose of SAN group (basal diet containing 2.25 mg/kg SAN). Values are expressed as mean ± SEM (*n =* 5). ∗*P* < 0.05 and ∗∗*P* < 0.01.

As indicated in Fig. 6A–C, dietary supplementation with different doses of SAN up-regulated the relative expression level of occludin in the cecum (*P* < 0.01). Furthermore, the Low and High groups showed significant upregulation of the mRNA levels of *ZO-1* (*P* < 0.05) and claudin-1 (*P* < 0.01). These findings suggest that dietary supplementation with SAN has a certain protective effect on the intestinal barrier function in the cecum. As depicted in Fig. 6D–I, the mRNA levels of *AvBD1* and *AvBD10* were increased (*P* < 0.01) in broilers receiving SAN compared to the CON group. In addition, the mRNA expression levels of *AvBD12* and *MUC2* were increased in the Low group (*P* < 0.01), and the expression of *TLR4* was decreased (*P* < 0.01). The Middle group demonstrated the expression level of *MUC2* was increased (*P* < 0.01), along with a significant decrease in *TLR4* expression (*P* < 0.05). Elevated *MUC2* mRNA expression (*P* < 0.05) and notable downregulation of *TLR2* mRNA expression (*P* < 0.05) were observed in the cecal tissues of the High group.

### 3.9 Effects of dietary SAN supplementation on cecal microbial structure and abundance in broilers

**Fig. 7:**
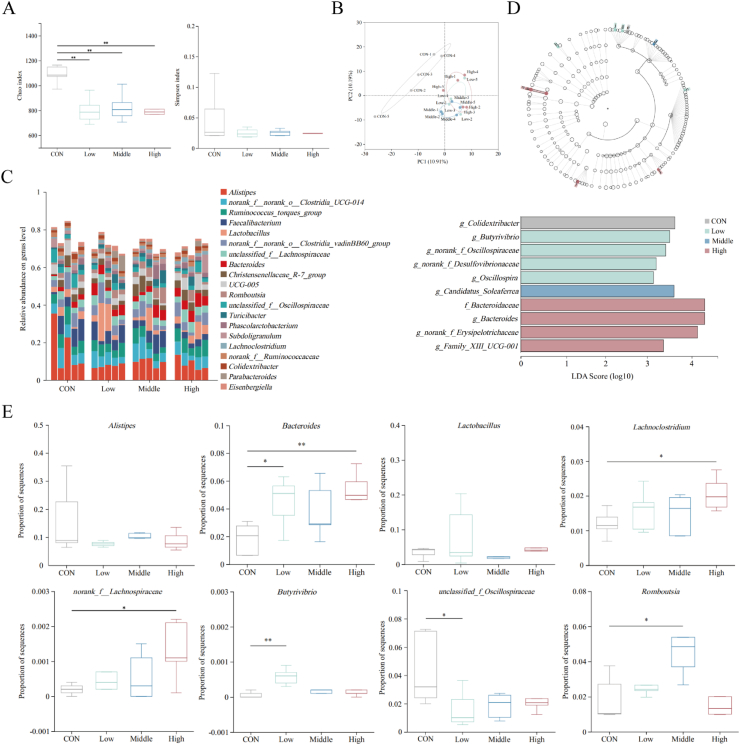
Effects of dietary sanguinarine (SAN) supplementation on the cecal microbiota of broilers. (A) The alpha diversity of the cecal microbiota of broiler chickens. (B) The beta diversity of the cecal microbiota of broilers was analyzed by PCA. (C) Top 20 microbes in the cecum of broilers at the genus level. (D) Taxonomic cladogram of bacteria from all groups and Linear Discriminant Analysis (LDA) scores of bacterial taxa that were significantly enriched in each group (LDA score >2). Different colors represent different groups and the length representing the LDA score which reflects the degree of effect of the significantly different species between groups. (E) Determination of different species of cecum microorganisms in different groups of broilers by T-test. CON, control group (basal diet); Low, low-dose of SAN group (basal diet containing 0.225 mg/kg SAN); Middle, medium-dose of SAN group (basal diet containing 0.75 mg/kg SAN); High, high-dose of SAN group (basal diet containing 2.25 mg/kg SAN). Values are expressed as mean ± SEM (*n =* 5). ∗*P* < 0.05 and ∗∗*P* < 0.01.

As depicted in Fig. 7A, compared with the CON group, the Simpson index of the SAN group was lower, but the difference was less pronounced (*P >* 0.05). The Chao index was decreased (*P* < 0.01), indicating that dietary supplementation of SAN altered the diversity of the cecal microbial community. As shown in Fig. 7B, based on PCA analysis, the samples from the CON group and SAN groups with different doses were widely dispersed among groups, indicating that dietary supplementation of SAN had a considerable influence on the composition of cecal microbiota (*P* < 0.05). As depicted in Fig. 7C, the addition of SAN to the diet increased the relative abundance of *Lactobacillus*, *Bacteroides* and *Collinsella* at the genus level, while decreasing the proportions of *UCG-005* and *Subdoligranulum*. As shown in Fig. 7D, the cecal microorganisms with notable discrepancies in each group of broilers were filtered using LDA with a threshold of 2. The Low group had increased abundances of *Butyrivibrio*, *norank_f__Oscillospiraceae*, *norank_f__Desulfovibrionaceae* and *Oscillospira* in the cecum, whereas for the Middle group, the relative abundance of *Candidatus_Soleaferrea* was considerably increased. Additionally, the High group exhibited a substantial increase the relative abundances of Bacteroidaceae, *Bacteroides*, *norank_f__Erysipelotrichaceae* and *Family_XIII_UCG-001* in the cecum. As shown in Fig. 7E, the cecal content samples were subjected to significance testing for the dominant bacteria genera which revealed that the abundance of *Bacteroides* (*P* < 0.05) and *Butyrivibrio* (*P* < 0.01) increased in the Low group, while the abundance of *unclassified_f__Oscillospiraceae* significantly decreased (*P* < 0.05). The Middle group exhibited a significant increase in the relative abundance of *Romboutsia* (*P* < 0.05). For the High group, there was a significant increase in the relative abundance of *Bacteroides* (*P* < 0.01), along with increased abundance of *Lachnoclostridium* and *norank_f__Lachnospiraceae* (*P* < 0.05).

### 3.10 Correlation between SCFA and immune-related factors and microbial structure

**Fig. 8:**
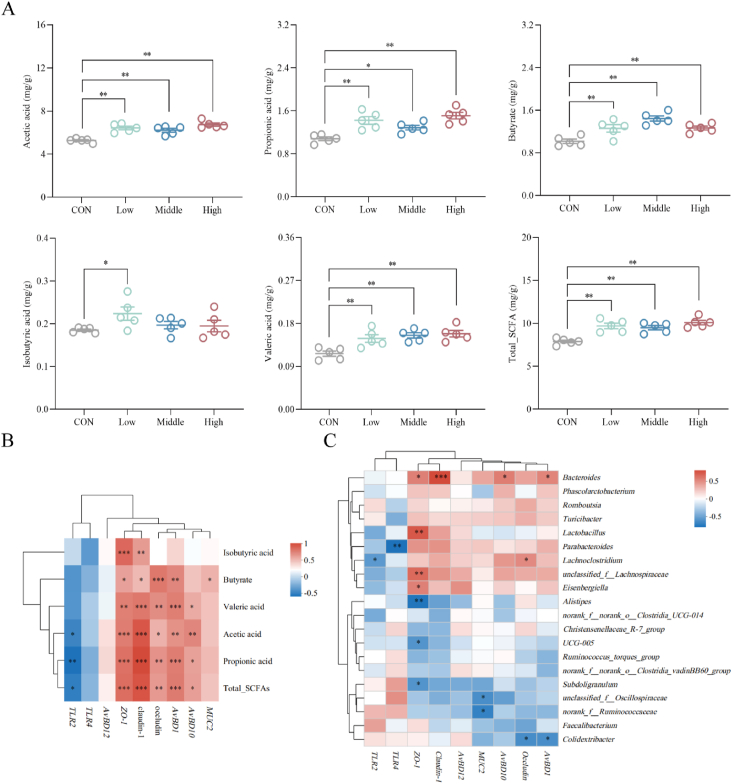
Correlation between short chain fatty acid (SCFA) and immune-related factors and microbial structure. (A) The concentration of various SCFA in the cecum was determined by gas chromatography. (B) Spearman correlation analysis was conducted to assess the correlation between SCFA and immune-related factors in the cecum. (C) Spearman correlation analysis was performed to evaluate the correlation between SCFA at the genus level and microbial taxa. *ZO-1* = zonula occludens-1; *AvBD1* = avian beta-defensin 1; *AvBD10* = avian beta-defensin 10; *AvBD12* = avian beta-defensin 12; *MUC2* = mucin 2; *TLR2* = Toll-like receptor 2; *TLR4* = Toll-like receptor 4. CON, control group (basal diet); Low, low-dose of SAN group (basal diet containing 0.225 mg/kg SAN); Middle, medium-dose of SAN group (basal diet containing 0.75 mg/kg SAN); High, high-dose of SAN group (basal diet containing 2.25 mg/kg SAN). Values are expressed as mean ± SEM (*n =* 5). ∗*P* < 0.05, ∗∗*P* < 0.01 and ∗∗∗*P* < 0.001. SAN = sanguinarine.

As shown in Fig. 8A, different doses of dietary SAN supplementation resulted in a significant increase in the concentrations of acetic acid, butyrate and valeric acid in the cecum (*P* < 0.01), and thus increased the total SCFA content. In addition, the content of propionic acid showed a highly significant increase in both the Low and High groups (*P* < 0.01), and a significant increase in the Middle group (*P* < 0.05). There was a significant increase in the content of isobutyric acid in the Low group (*P* < 0.05). Additionally, correlation analysis was performed to assess the relationship between SCFAs and cecum microbiota. As represented in Fig. 8B, a positive correlation was observed between acetic acid, propionic acid, valeric acid, total SCFAs, and *ZO-1*, occludin, claudin*-*1, *AvBD1*, and *AvBD10*, the contents of butyrate were found to be positively correlated with *ZO-1*, occludin, claudin-1, *AvBD1* and *MUC2*. Conversely, acetic acid, propionic acid and total SCFAs showed a negative correlation with *TLR2*. Additionally, correlation analysis was performed to assess the relationship between SCFAs and cecal microbiota. As illustrated in Fig. 8C, *Bacteroides* showed a positive correlation with the production of SCFAs, while *Lactobacillus* exhibited a positive correlation with the production of propionic acid, isobutyric acid and total SCFAs. Additionally, *unclassified_f__Lachnospiraceae* demonstrated a positive correlation with propionic acid, butyrate and total SCFAs. Conversely, the production of butyrate exhibited a negative correlation with *Colidextribacter*.

## 4 Discussion

An increasing number of studies have demonstrated that the addition of MCE to the diet has a favorable effect on animal growth performance. For example, supplementing the diet with MCE has the potential to enhance the growth performance of early-weaned piglets (Jiashun et al., 2018). Incorporating MCE into the diet at concentrations of 15, 50 and 150 mg/kg resulted in a significant increase in the feed intake of broilers; furthermore, the addition of 50 mg/kg MCE also led to a significant reduction in the feed conversion ratio of broilers throughout the entire feeding period (Huang et al., 2018). Sanguinarine, one of the main monomeric components of MCE, exhibits a growth-promoting effect similar to that of MCE when added to feed. Previous studies have reported that incorporating 1.5% SAN into the diet regulates serum cholesterol levels and significantly improves growth performance and meat quality (Lee et al., 2015; Vieira et al., 2008). Our study showed that supplementing diets with SAN increased the FCR from 22 to 42 d and 1 to 42 d, which might be associated with SAN's role in regulating serum biochemical metabolism in broilers. Therefore, we further evaluated the impact of SAN on the serum biochemical parameters in broilers.

Serum biochemical parameters are crucial indicators of health status and metabolic alterations (Wang et al., 2022a). Aminotransferase, AST and ALP are markers of liver damage (Jia et al., 2021) and elevated levels of serum ALT, AST and ALP are closely associated to excessive inflammation (Feng et al., 2020; Nasrollahi et al., 2019). Our data showed a significant reduction in the levels of ALT and ALP with the inclusion of varying doses of SAN in the diet. In addition, the Low group had reduced AST, indicating that the addition of SAN in the broiler diet had a positive effect on liver function and may even mitigate the risk of liver inflammation to a certain extent. The concentrations of TP and ALB are indicators for assessing protein digestion, absorption and immune function in the body. The levels of BUN, UA and CREA reflect protein metabolism and amino acid balance in animals (Lee et al., 2015). The administration of 0.7 mg/kg of SAN to the diet up-regulated the level of TP in the serum of broilers to improve protein synthesis efficiency (Chashnidel et al., 2020; Liu et al., 2020). In our study, the inclusion of SAN in the diet resulted in a significant increase in TP and ALB levels, as well as a decrease in UA, BUN and CREA levels. These findings suggested that SAN had a positive impact on protein synthesis, conversion and deposition in broilers. Creatine kinase and LDH are enzymes associated with myocardial injury. Studies have shown that under heat stress, increased myocardial cell membrane permeability and a series of enzymes are released outwards, resulting in increased levels of CK and LDH in serum (Wu et al., 2015). As demonstrated in this study, the levels of CK and LDH in serum were reduced with the inclusion of SAN in the diet. The trend of changes in serum biochemical parameters is consistent with broiler growth performance, indicating that SAN can promote protein digestion and absorption, regulate amino acid metabolism and improve growth performance. It is hypothesized that the effect of SAN on serum biochemical parameters in broilers is related to its protective effect on intestinal barrier function. Consequently, we next proceeded to assess the effect of SAN on indicators related to intestinal barrier function in broilers.

The growth performance of broilers relies heavily on a healthy intestinal system, including a normal physiological state, optimal digestion and absorption function, immune function and a balance of intestinal microbiota (Song et al., 2023). The intestinal morphological structure and tight junction protein expression are often utilized to assess intestinal injury (Song et al., 2022). Occludin and claudins serve as key constituents of intestinal tight junctions, exerting control over the barrier properties of the paracellular space through the formation of tight junction chains. ZO-1 plays a crucial role that connects transmembrane proteins with intracellular actin in regulating intestinal tight junctions (Turner, 2009). Dietary MCE supplementation at 120 mg/kg can reduce the intestinal damage score of NE broilers (Hussein et al., 2020). Sanguinarine could increase serum IgG levels and villus height, and improve immune response in broilers (Zhang et al., 2022). The expression of intestinal barrier genes can be enhanced by administering SAN via gavage at a dosage of 1 mg/kg, thus enhancing the function of the intestinal barrier (Lin et al., 2022). The addition of SAN to the diet has been shown to effectively decrease crypt depth, enhance the value of V/C, and subsequently improve intestinal morphology (Abudabos et al., 2019; Liu et al., 2020). In our study, the addition of SAN to the diet can not only increase the villus height and V/C values in the ileum and reduce crypt depth, but also up-regulate the mRNA expression of genes related to intestinal barrier function in both the ileum and cecum. These findings suggest that SAN can improve intestinal barrier function and maintain normal intestinal morphology. We hypothesized that SAN may exert a protective effect on the intestinal barrier through its biological activity. In addition to its possible antibacterial activity, it may also indirectly improve intestinal barrier function by regulating immune function. Consequently, we conducted a further investigation into the impact of dietary SAN supplementation on the immune system of broilers.

The intestine serves as a major immune organ in the body with the functions of defense, monitoring and self-regulation (Xue et al., 2022). B-cell differentiation and proliferation can be stimulated by IL-4 and IL-10, increasing immunoglobulin content, thus mediating humoral immunity. MUC2, the primary protective mucin in the mucus layer, is secreted by goblet cells to form a gel-like barrier. Intestinal antimicrobial peptides (AvBD1, AvBD10 and AvBD12) are vital components to defend the mucosa against damage and maintain intestinal health (Cheng et al., 2022). Toll-like receptors activate immune responses and host defense, and binding to pathogen-associated patterns initiates specific signaling pathways, activating NF-κB and IL-1β production, thus enhancing immune response (Ashley et al., 2012; Vajjhala et al., 2017). Sanguinarine can significantly up-regulate the mRNA level of *IL-10* in cecal tissue, increase the content of serum IgA and IgG, and reduce the content of serum IL-1β (Song et al., 2023; Zhang et al., 2022). The addition of baicalin can increase the expression levels of *MUC2* and *AvBDs*, and alleviate intestinal damage induced by *E. coli* (Cheng et al., 2022). In this study, we found that dietary addition of SAN upregulated the levels of *IgA*, *IgM*, *IgG*, *IL-4*, *IL-10* and *IFN-γ* in serum and ileal tissue. Moreover, it resulted in a notable increase in the relative mRNA expression levels of *AvBD1*, *AvBD10*, *AvBD12* and *MUC2* in ileal and cecal tissue. The mRNA levels of *TLR2* and *TLR4* were decreased, and the protein expression contents of *TLR4*, *IL-1β* and *NF-κB* were also reduced in the ileum. Therefore, we speculated that SAN has the potential to enhance immune function by reducing the levels of pro-inflammatory cytokines and increasing the production of anti-inflammatory and defense factors in both serum and intestinal tissue. In addition, we acknowledge the close relationship between intestinal immune function and the composition and abundance of the microbial population. Due to its antibacterial activity, SAN can effectively inhibit the proliferation of certain pathogenic bacteria within the intestine, ultimately leading to a decrease in metabolite production by these harmful bacteria and preserving the integrity of the intestinal mucosa. Consequently, our further research focused on exploring the influence of SAN on the microbial structure and abundance in the cecum of broilers.

The gut microbiota, the largest symbiotic ecosystem within the host's body, plays a vital role in the development and maturation of the mucosal immune system. The presence of a healthy gut microbiota can regulate the maturation of the mucosal immune system, consequently enhancing the host's resistance against potentially harmful pathogenic bacteria. Conversely, pathogenic bacterial communities can utilize sources of carbon and nitrogen derived from the microbiota to fuel their own growth and produce toxins, and create an environment that is conducive to their multiplication, thereby interfering with immune function and causing immune dysfunction and disease (Baumler et al., 2016; Na et al., 2017). Early colonization and establishment of the gut microbiota can improve intestinal morphology and physiological functions in chickens by reducing sensitivity to external pathogens (Kers et al., 2018; Wang et al., 2016). Research has indicated that during heat stress conditions, the cecum of broilers experiences a significant increase in the relative abundance of *Clostridium perfringens* and *E. coli*. Meanwhile, there is a notable decrease in the relative abundance of beneficial bacteria such as *Lactobacillus*, *Bifidobacterium* and *Phaseolus acutifolius*, as well as a significant decline in the overall α-diversity of the jejunal microbiota (Yang et al., 2022). Our data showed that incorporating various doses of SAN into the diet of broiler chickens contributed to modulations in the diversity of their cecal microbiota. The Chao index and Simpson index both decreased, which might be attributed to the targeted regulation of the abundance of certain bacterial groups by SAN. β-diversity analysis also confirmed that feeding different doses of SAN can regulate the structural composition of the cecal microbiota in chickens. Based on the previous studies, *Lactobacillus*, *Butyrivibrio* and *Bacteroides* are identified as important bacteria for SCFA production (Huang et al., 2018; Kaakoush et al., 2014; Yu et al., 2024). Short chain fatty acids play crucial roles in maintaining intestinal physiology. They help preserve the integrity of the intestinal mucosa and protect the intestine from inflammatory damage (Bartolomaeus et al., 2019; Wang et al., 2022c). Numerous studies have shown that acetate and butyrate have the greatest impact on host health. Acetate can lower the incidence of cardiovascular diseases by inducing changes in relevant genes through increased acetate levels, thus improving cardiovascular health (Marques et al., 2017). Butyrate not only acts as an inhibitor of virulence factor expression in bacterial pathogens, but also as an energy substrate for colonic epithelial cells (Boyen et al., 2008). This study found that adding SAN to the diet led to a higher proportion of *Lactobacillus*, *Butyrivibrio* and *Bacteroides* in the cecum. Other research has shown that phage CKT1 can effectively decrease *Shigella* population, which tends to increase due to *Salmonella* invasion in the intestines. Additionally, it can also promote the growth of beneficial microbiota like *Lachnoclostridium*, *Ruminococcus*, *Lactobacillus* and *Pseudoflavonifractor (*Huang et al., 2022). Feeding broilers with a *Bacillus subtilis* supplement in their diet can enhance the proportion of *Ruminococcus*, *Lachnoclostridium* and *Anaerostipes* in the intestinal tract, thereby promoting the production of beneficial metabolites, such as butyrate, improving intestinal structure and promoting growth performance in broilers (Jacquier et al., 2019). The increased abundance of SCFA-producing bacteria not only reduces the environment's pH value and inhibits the colonization of pathogenic bacterial communities, but also adjusts the relative expression levels of cecal immune-related factors and improves intestinal health. Research has shown that feeding buckwheat polysaccharides can increase the production of SCFAs, and it is possible to decrease the presence of inflammatory bacteria, such as *Oscillospiraceae* and *Oscillibacter*, thereby improving TNBS-induced colitis (Yang et al., 2023). Painong-San extract can down-regulate the expression of inflammatory proteins in the intestine, inhibit the *TLR4*/*NF-κB* signaling pathway, regulate intestinal flora disturbance, suppress the excessive proliferation of conditional pathogenic bacterial communities such as *Oscillospiraceae* and *Helicobacter*, increase the relative abundance of *Romboutsia*, *Lactobacillus* and *Akkermansia*, and relieve colon inflammatory response (Wang et al., 2022b). Previous research findings have indicated that dietary MCE supplementation primarily affects the microflora composition in the small intestine of broilers. It has been observed to enhance the growth of *Lactobacillus* while inhibiting the colonization of *E. coli* (Huang et al., 2018). Additionally, it was discovered that feeding weaned piglets with 1.5% SAN supplementation led to a significant rise in the proportion of *Lactobacillus* in both the ileum and cecum. The supplementation also stimulated the production of propionic acid, butyric acid and SCFAs, while simultaneously decreasing the presence of *E. coli* and *Salmonella* in both the ileum and cecum (Chen et al., 2018). In our study, SAN substantially elevated the proportion of bacteria (*Bacteroides*, *Butyrivibrio*, *unclassified_f__Lachnospiraceae*, *Lactobacillus* and *Romboutsia*) that produce SCFAs in the cecum. It also decreased the proportion of bacteria associated with inflammation (*UCG-001* and *Spirillaceae*), promoted the production of beneficial metabolites, and enhanced intestinal immune function, thus improving intestinal health.

## 5 Conclusion

In summary, SAN, as a key active component in MCE, can enhance intestinal immune function, target the structure and abundance of the gut microbiota, improve gut health and enhance the growth performance of broiler chickens.

## Credit Author Statement

**Yue Su:** Conceptualization, Formal analysis, Investigation, Data curation, Writing - original draft. **Guanyu Chang** and **Jingyu Liu:** Conceptualization, Investigation, Methodology, Writing e review & editing. **Peng Huang** and **Jianguo Zeng:** Conceptualization, Writing - review & editing, Supervision, Funding acquisition.

## Declaration of competing interest

We declare that we have no financial and personal relationships with other people or organizations that can inappropriately influence our work, and there is no professional or other personal interest of any nature or kind in any product, service and/or company that could be construed as influencing the content of this paper.
