# Supplementary material for: Dietary sanguinarine supplementation improves the growth performance and intestinal immunity of broilers
Source: Anim Nutr. 2024 Aug 12;19:76–89. doi: 10.1016/j.aninu.2024.05.009 (PMC11605185; doi:10.1016/j.aninu.2024.05.009)
Supplement: Multimedia component 1 [file mmc1.docx]

Table S1 Primers used for real-time quantitative PCR^1^.

| Gene^2^ | Primers^3^ (5’→3’) | GenBank accession |
| --- | --- | --- |
| β-Actin | F: GCACCACACTTTCTACAATGAG | NM_205518.2 |
|  | R: ACGACCAGAGGCATACAGG |  |
| *ZO-1* | F: GCCAGCCATCATTCTGACTCCAC | XM_046925214.1 |
|  | R: GTACTGAAGGAGCAGGAGGAGGAG |  |
| Occludin | F: TACGGCAGCACCTACCTCAA | XM_046904540.1 |
|  | R: AGGCAGAGCAGGATGACGAT |  |
| Claudin-1 | F: GCCACGTCATGGTATGGCAA | NM_001013611.2 |
|  | R: CCAGCCAATGAAGAGGGCTG |  |
| *AvBD1* | F: CCCTTCATCCTCCTCCTGGC | NM_204993.1 |
|  | R: TGATGAGAGTGAGGGAAGGGC |  |
| *TLR2* | F: ACATGTGTGAATGGCCTGAA | XM_046915414.1 |
|  | R: TTGAGAAATGGCAGTTGCAG |  |
| *MUC2* | F: ATGCGATGTTAACACAGGACTC | XM_040673077.2 |
|  | R: GTGGAGCACAGCAGACTTTG |  |
| *AvBD10* | F: CTGCTCTTCGCTGTTCTCCT | NM_001001609.3 |
|  | R: GGTGTCAGGGAAAAGTGGGT |  |
| *AvBD12* | F: AACCACGACAGGGGATTGTG | NM_001397753.2 |
|  | R: CAGGTCTTGGTGGGAGTTGG |  |
| *TLR4* | F: ACGGCATTTCAGAACGGACT | NM_001030693.2 |
|  | R: GGGCTTGGAGTGGCTTGTAT |  |

^1^ Primers were designed using Primer Express software (Sangon Biotech, Shanghai, China).

^2^ *ZO-1* = zonula occludens-1; *AvBD1* = avian beta-defensin 1; *TLR2* = Toll like receptor 2; *MUC2* = mucin 2; *AvBD10* = avian beta-defensin 10; *AvBD12* = avian beta-defensin 12; *TLR4* = Toll like receptor 4.

^3^ F, Forward; R, Reverse.
